# Supplementary material for: Probiotics (Lactobacillus plantarum HNU082) Supplementation Relieves Ulcerative Colitis by Affecting Intestinal Barrier Functions, Immunity-Related Gene Expression, Gut Microbiota, and Metabolic Pathways in Mice
Source: Microbiol Spectr. 2022 Nov 2;10(6):e01651-22. doi: 10.1128/spectrum.01651-22 (PMC9769980; doi:10.1128/spectrum.01651-22)
Supplement: Supplemental file 1 — Supplemental material. Download spectrum.01651-22-s0001.pdf, PDF file, 1.9 MB [file spectrum.01651-22-s0001.pdf]

Supplementary figure legends

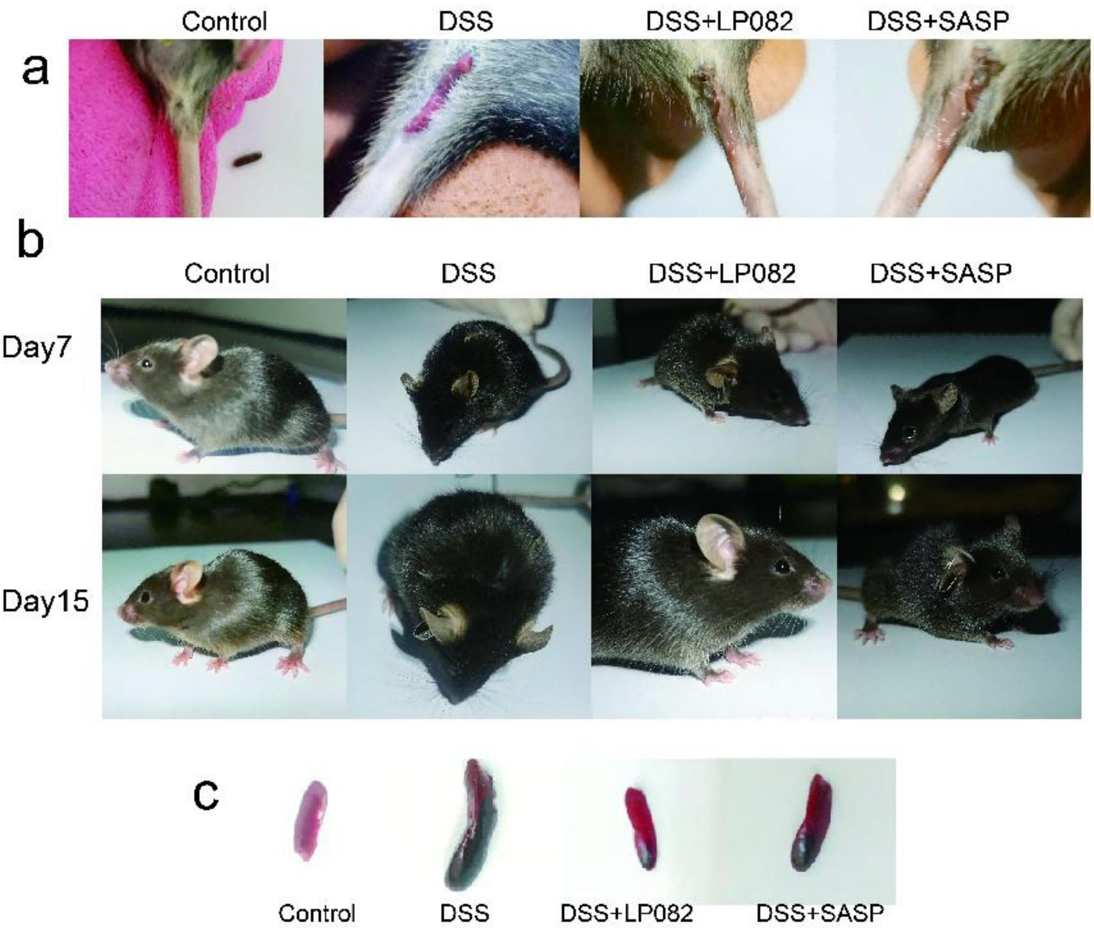

Fig. S1.

(a) Blood in the stool of experimental mice.

(b) Mental state of experimental mice.

(c) Spleen condition of experimental mice.

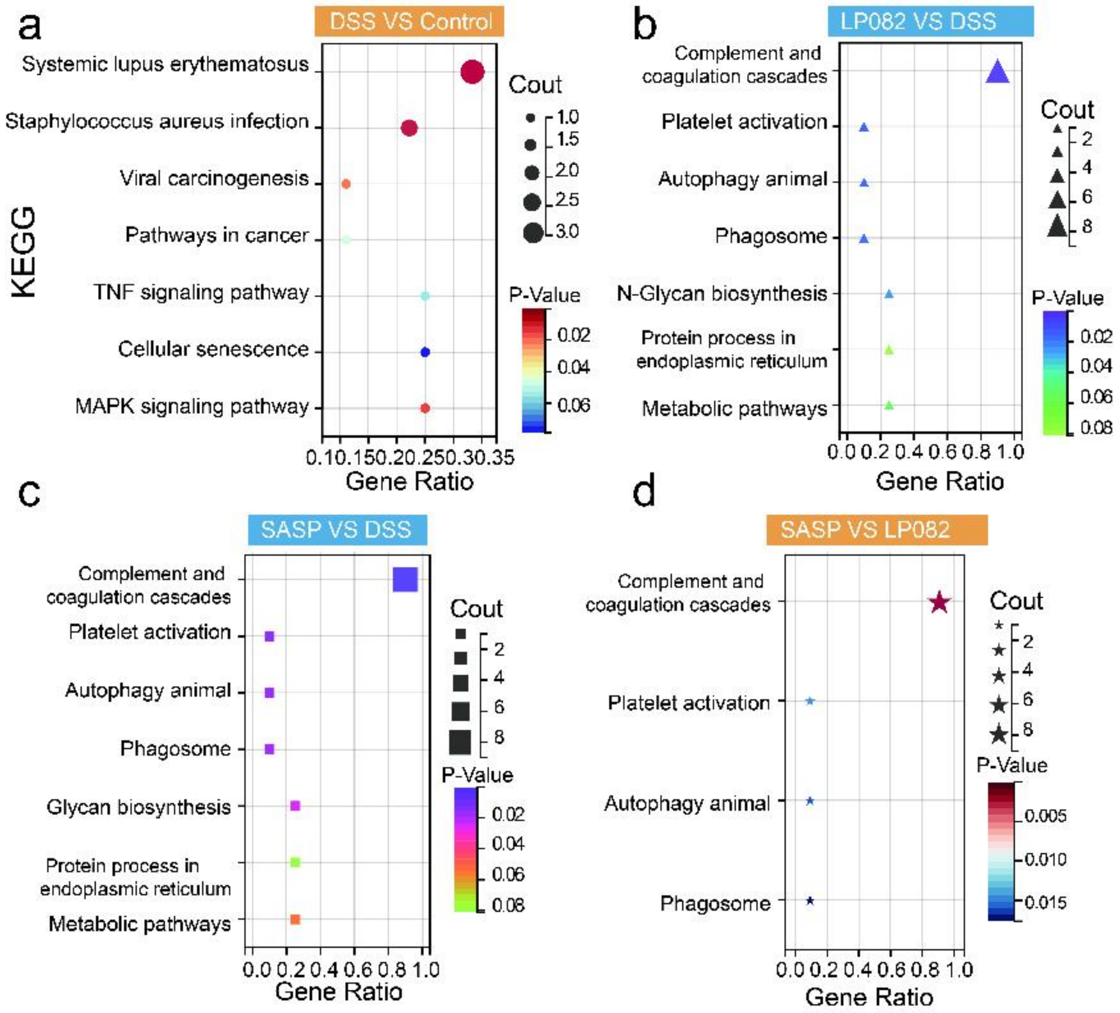

Fig. S2.

(a) KEGG pathway enriched by differentially expressed genes between DSS and control groups.

(b) KEGG pathway enriched by differentially expressed genes between Lp082 and DSS groups.

(c) KEGG pathway enriched by differentially expressed genes between SASP and DSS groups.

(d) KEGG pathway enriched by differentially expressed genes between SASP and Lp082 groups.

Wilcoxon signed-rank test is used here. Each group had at least 6 biological replicates.

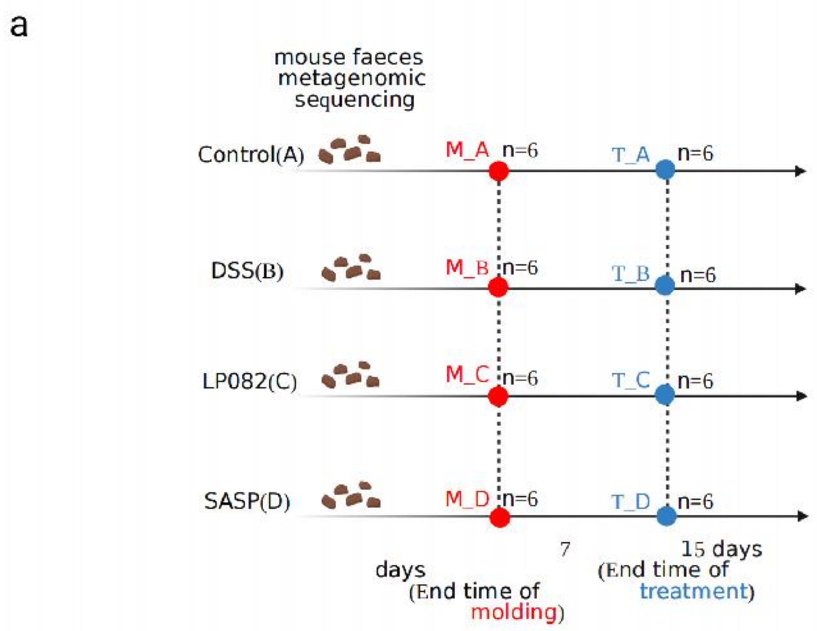

Fig. S3 (a) Timepoints and grouping of mouse metagenomic sequencing

M means the modeling period, T means the treatment period. Respectively, A, B, C and D group mean 7 days normal water (ultrapure water), DSS, Lp082 and SASP treatment after 7 days DSS gavage.

M-A means A group represents the control group on the 7th day of DSS modeling, M-B represents the DSS group on the 7th day of DSS modeling, M-C represents the Lp082 group on the 7th day of DSS modeling, M-D represents the SASP on the 7th day of DSS treatment Group.

T-A means treating-A group represents the control group at the end of the treatment, T-B represents the DSS group at the end of the treatment, T-C represents the Lp082 group at the end of the treatment, and T-D represents the SASP group at the end of the treatment.

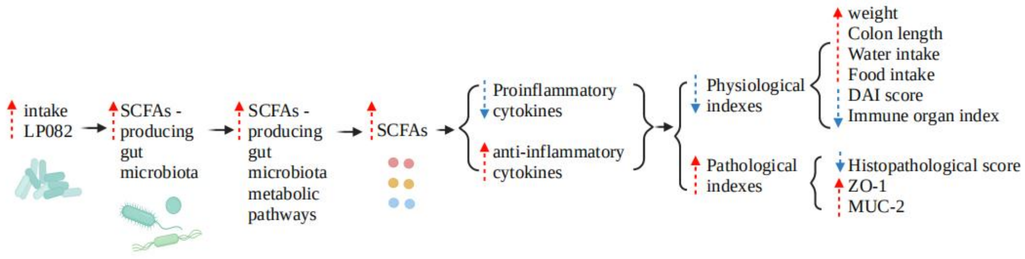

Fig. S4. The underlying mechanism by which Lp082 regulates SCFAs to alleviate UC

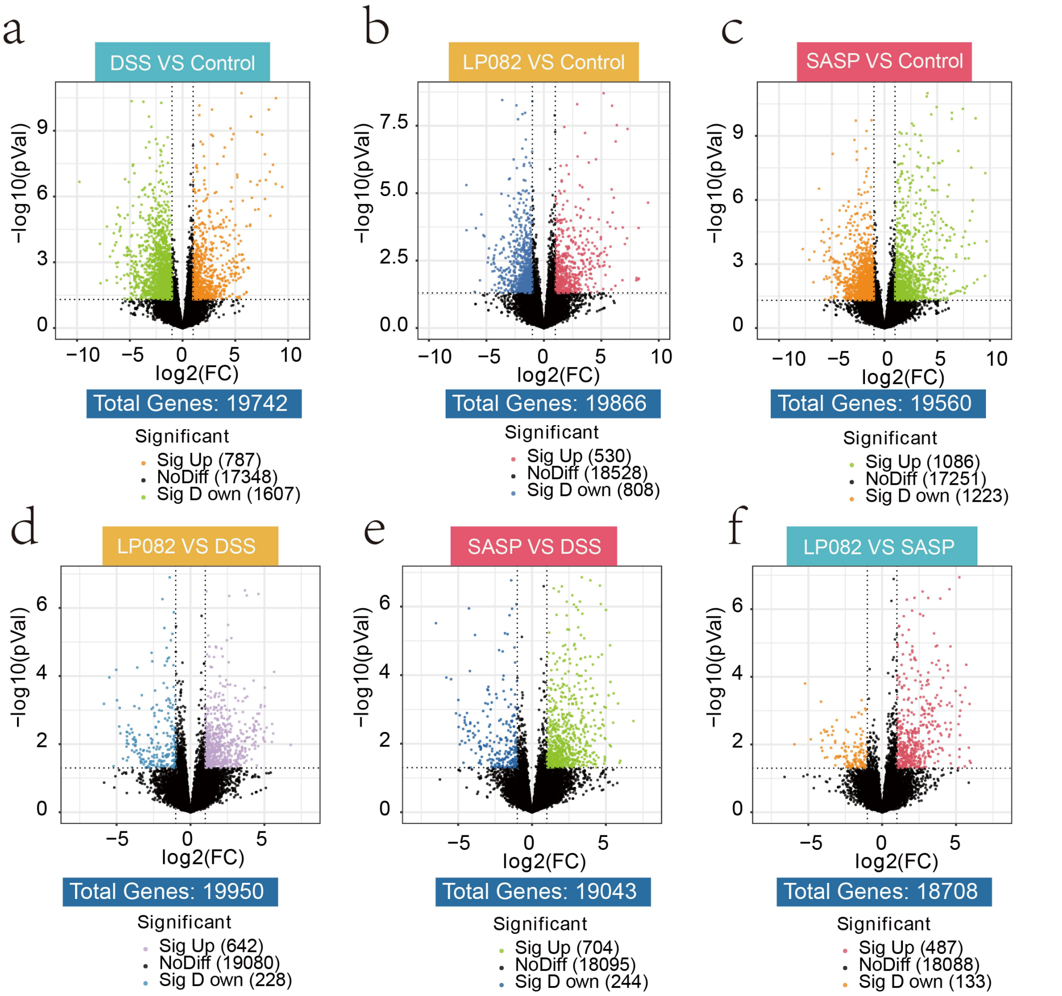

Fig. S5. Effects of Lp082 on gene expression distribution in mouse colon tissue.

(a)Gene expression distribution in the DSS group compared with the control group.

(b)Gene expression distribution in the Lp082 group compared with the control group.

(c)Gene expression distribution in the SASP group compared with the control group.

(d)Gene expression distribution in the Lp082 group compared with the DSS group.

(e)Gene expression distribution in the SASP group compared with the DSS group.

(f)Gene expression distribution in the Lp082 group compared with the SASP group.

Wilcoxon signed-rank test is used here. Each group had at least 6 biological replicates.

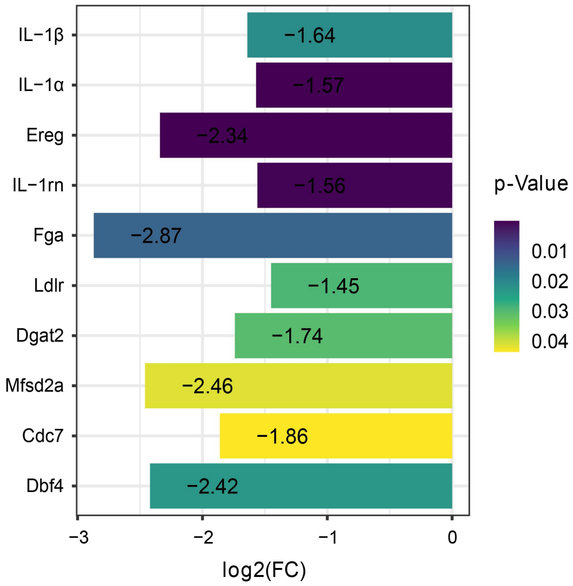

Fig. S6. The effect of Lp082 treatment on up-regulated inflammatory genes in the DSS group in Fig. 6d.

The 13 inflammatory genes or oncogenes that were up-regulated and enriched in the inflammatory pathway in the DSS group, the following 10 genes were significantly down-regulated in the Lp082 group: IL-1β, IL-1α, Ereg, IL -1rn, Fga, Ldlr, Dgat2, Mfsd2a, Cdc7, Dbf4.

Wilcoxon signed-rank test is used here. Each group had at least 6 biological replicates.

Supplementary table legends

Score	Weight loss (%)	Stool consistency	Visible blood in faeces
0	0	Normal	Normal
1	1~5	Loose state, which is pasty and semi-formed stool that does not adhere to anus	Positive for FOBT
2	6~10	Obvious loose state, which is a pasty and basically unformed stool that does not adhere to the anus	Strongly positive for FOBT
3	11~15	Dilute stool is a watery stool that can adhere to the anus	Visible blood in faeces
4	≥16	The degree of loose stool is more obvious, and the thin water sample is more clear and long	Obvious visible blood in faeces

Table S1.

Disease activity index (DAI) scoring system of dextran sodium sulfate-induced colitis. The DAI scoring system consists of three parts: weight loss, stool consistency, and visible blood in feces. Each part has 5 grades from 0 to 4. A score of 0 means that the three indicators are normal, and the closer the score is to 4, the more serious inflammation it is.

Project	Degree	Score
**Inflammation**	None	0
	Mild or few	1
	Moderate	2
	Severe massive	3
	Extremely severe and massive	4
**Depth of inflammation**	None	0
	Submucosal layer	1
	muscular layer	2
	Serous layer	3
	Transmural	4
**Range of inflammation (%)**	0	0
	1~25	1
	26~50	2
	51~75	3
	76~100	4
**Cryp damage**	None	0
	1/3 crypt of basement was destroyed	1
	2/3 crypt of basement was destroyed	2
	Only intact surface epithelium	3
	All crypts and epithelium were destroyed	4
**Goblet cell loss**	None	0
	1~25%	1
	26~50%	2
	51~75%	3
	75~100%	4

**The degree of neutrophil infiltration**	None	0
	Crypt base infiltration	1
	Muscularis mucosae infiltrate	2
	Muscularis mucosae severity infiltrate	3
	Mucosal thickening and edema	
	Submucosal infiltration	4

Table S2.

Histopathology scoring system of dextran sodium sulfate-induced colitis. The Histopathology scoring system was modified from previous studies [70]. The modified scoring system consists of six parts, namely, depth of Inflammation, range of inflammation (%), crypt damage, goblet cell loss, and the degree of neutrophil Infiltration. Each component was rated on a scale of 0 to 4, a score of 0 means that the three indicators are normal, and the closer the score is to 4, the more serious inflammation it is.
